# Poole–Frenkel (PF)-MOS: A Proposal for the Ultimate Scale of an MOS Transistor

**DOI:** 10.3390/nano13030411

**Published:** 2023-01-19

**Authors:** Hei Wong, Kuniyuki Kakushima

**Affiliations:** 1 Department of Electrical Engineering, City University of Hong Kong, Hong Kong, China; 2 Frontier Research Center, Tokyo Institute of Technology, Yokohama 226-8502, Japan

**Keywords:** lateral Poole–Frenkel emission, interface, PF-MOS, nano CMOS

## Abstract

This work reports, for the first time, the phenomenon of lateral Poole–Frenkel current conduction along the dielectric/Si interface of a silicon nanowire metal-oxide semiconductor (MOS) transistor. This discovery has a great impact on the study of device characteristic modeling and device reliability, leading to a new kind of electronic device with a distinct operation mechanism for replacing the existing MOS transistor structure. By measuring the current–voltage characteristics of silicon nanowire MOS transistors with different nanowire widths and at elevated temperatures up to 450 K, we found that the current level in the conventional ohmic region of MOS transistors, especially for the transistors with a nanowire width of 10 nm, was significantly enhanced and the characteristics are no longer linear or in an ohmic relationship. The enhancement strongly depended on the applied drain voltage and strictly followed the Poole–Frenkel emission characteristics. Based on this discovery, we proposed a new type of MOS device: a Poole–Frenkel emission MOS transistor, or PF-MOS. The PF-MOS uses the high defect state Si/dielectric interface layer as the conduction channel and is expected to possess several unique features that have never been reported. PF-MOS could be considered as the ultimate MOS structure from a technological point of view. In particular, it eliminates the requirement of a subnanometer gate dielectric equivalent oxide thickness (EOT) and eradicates the server mobility degradation issue in the sub-decananometer nanowires.

## 1. Introduction

Although the CMOS technology is still advancing according to Moore’s Law in terms of chip density, it is assumed that the device downsizing will come to an end very soon, not because of the physical limit of device operation, but because of manufacturing issues such as the gate patterning, gate dielectric scaling, and the non-ideal issues arising from the surface and interface of the silicon nanoscale channel and the silicon gate dielectric interface [1,2,3,4]. The three-dimensional device structure, such as the FinFET and nanosheet transistor, has demonstrated quite promising capability for further reducing the device size by folding a large gate width along the boundaries to achieve a smaller footprint [4,5,6,7,8,9]. This structure has additional advantages in suppressing the threshold voltage roll-off, subthreshold conduction, and drain-induced barrier lowering (DIBL) [10,11]. As the fin width and sheet height became smaller, the surface roughness and interface state density could take a significant share in the current conduction and the inversion charge generation. Depending on the structures and parameters of the device, the bulk current conduction could be completely dismissed if the nanowire size is less than 5 nm, or even larger, in some cases [4,12,13]. Under this situation, the conduction current will be dominated by surface conduction. Hence, the minimum achievable gate length is not only governed by the lithography limit, but also by the minimum size of the bulk type channel and the best achievable oxide equivalent oxide thickness (EOT) of the gate dielectric. The reported minimum EOT thus far is around 0.5 nm, but the actual EOT used in the state-of-the-art technology was only 0.7 to 0.8 nm, which has already lagged behind the technology development for many generations [2,12]. Without a smaller EOT, the optimal device structures cannot be achieved. These two issues are difficult to overcome, and they will become the technology limits for further device downsizing.

We have attempted to tackle these issues. We found that even the gate EOT scaling is difficult. Even though the ~0.5 nm EOT is possible with high-dielectric constant (high-k) metal oxide deposited with the most advanced fabrication facility [3,14], the high-k/Si interfaces were found to have much higher interface state density, and the interface is less thermally stable, exhibiting a higher degree of surface roughness. In studying the sizing effect of the nanowire transistor, significant mobility degradation and some anomalous behaviors were found as the sizes of the nanowire were reduced to smaller than 10 nm [15]. In this report, we conduct a comprehensive study on the temperature-dependent current–voltage characteristics for different sizes of nanowire transistors and discover a new current conduction mechanism between the source and drain. As will be discussed in detail in Section 3, significant lateral field-dependent conduction was observed in the ohmic region of the MOS transistor. This observation is attributed to the Poole–Frenkel emission of the localized interface states along the channel direction. With this new lateral PF conduction regime, a new type of device operation, Poole–Frenkel MOS, or PF-MOS, is proposed, which will be illustrated in Section 4. This new device structure can eliminate the constraint of a minimum nanowire thickness, as well as the need for sub-nanometer gate dielectric EOT. It could be considered the ultimate structure of an MOS device.

## 2. Sample Preparation

**Figure 1:**
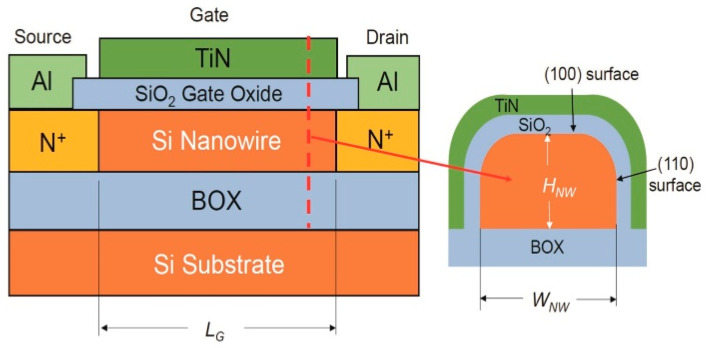
The nanowire transistor structure fabricated for this investigation.

It was reported that the nanosheet and nanowire transistors usually have smaller channel mobility than the conventional types [6,7,8,9,10,11], and the mobilities were also found to be size-dependent [2]. Here, we conducted further comprehensive experimental measurements on the sizing effects of the nanowire transistors. The nanowire transistors used in this investigation were fabricated in Iwai/Kakushima’s Lab at the Tokyo Institute of Technology, Japan. The starting material was an SOI wafer with a buried oxide (BOX) layer. Silicon fins of about 15 nm high with widths varying from 10 nm to 130 nm were patterned using a silicon nitride mask. To provide a large drain current, each device consisted of 100 nanowires of the same size. The source and drain (S/D) regions were patterned with a thermally-grown oxide layer, and their doping was performed with phosphorus implantation with a dose of 3 × 10^14^ cm^−2^. After the post-implant annealing at 800 °C, the TiN gate electrode was deposited and patterned. Forming gas (with 3% H_2_) annealing was conducted at 420 °C. The device structure is depicted in Figure 1. To avoid possible short-channel effects, the devices used in this investigation had a long gate length of 1.5 μm.

## 3. Results and Discussion

**Figure 2:**
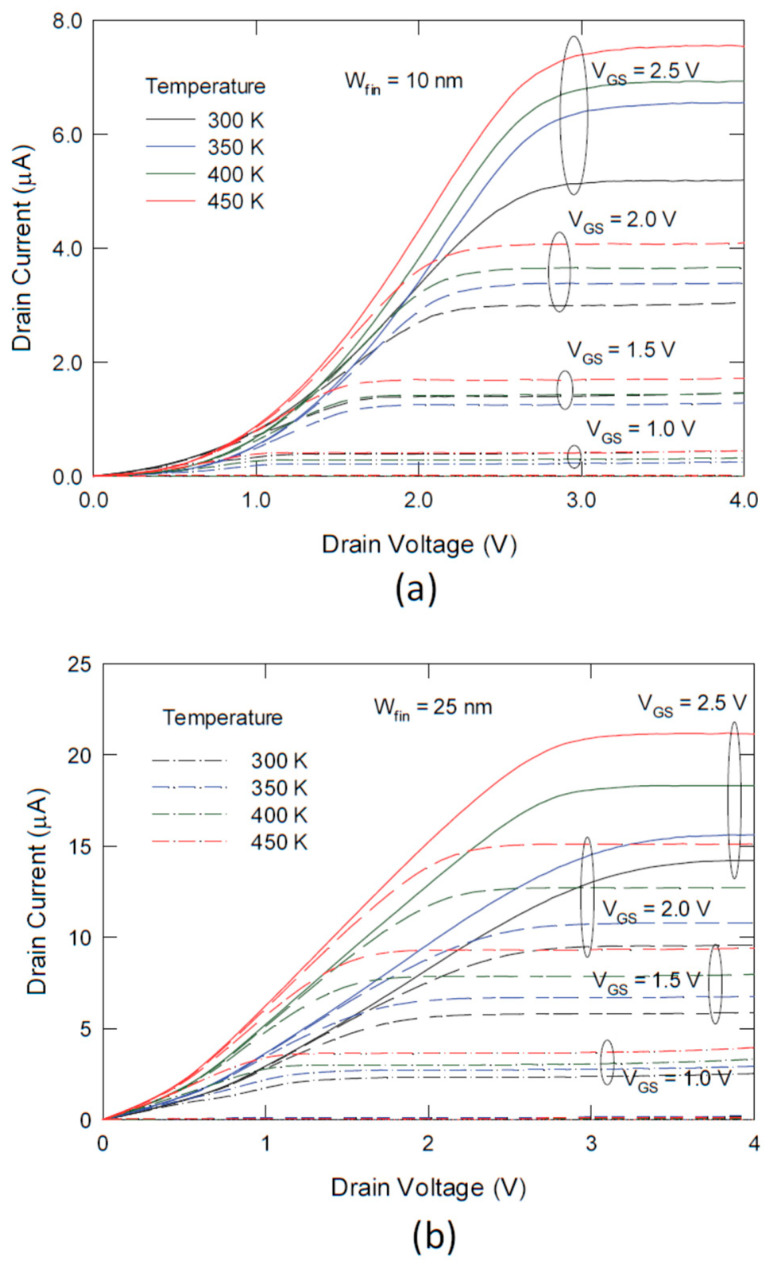
Plot of the current–voltage characteristics of nanowire transistor at different temperatures for nanowire widths of: (**a**) 10 nm, and (**b**) 25 nm. The gate length of the transistors was 1.5 μm.

Figure 2 compares the current–voltage characteristics of different nanowire size transistors: (a) 10 nm, and (b) 25 nm, for temperatures ranging from 300 K to 450 K. It is noted that for a nanowire width of *W_NW_* = 10 nm, highly nonlinear characteristics were found in the region of *V_DS_* < *V_GS_* − *V_T_*, i.e., the linear or ohmic region in the conventional MOS device regime. The drain current increased more rapidly as the drain voltage increased. Data analysis also shows that the saturation characteristics of this transistor were also diverted significantly from the (*V_GS_* − *V_T_*)^2^ relationship, which is the conventional I-V characteristic [16]. For *W_NW_* = 25 nm, the superlinear region became less obvious, but it was still notable, especially for a larger gate voltage and at a higher temperature.

**Figure 3:**
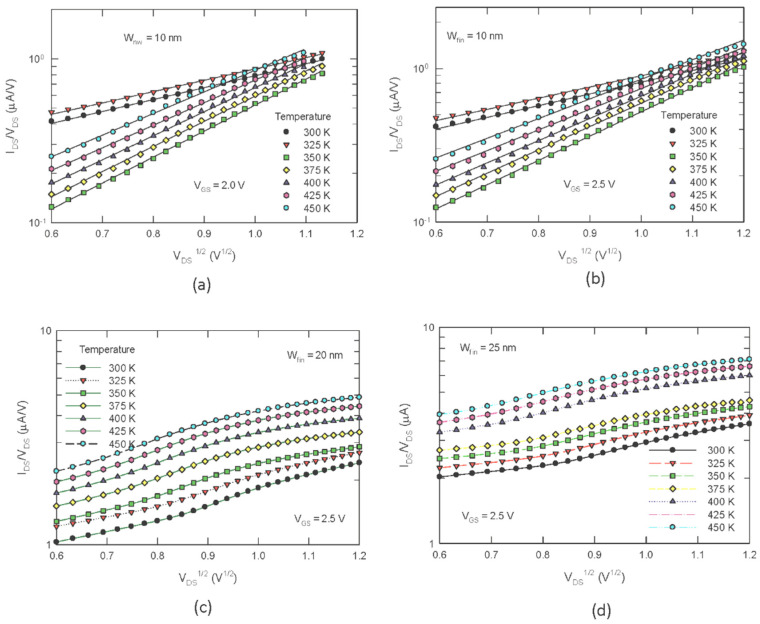
Poole–Frenkel plot of the superlinear drain current behaviors in the “ohmic region” at different temperatures: (**a**) nanowire width *W_NW_* = 10 nm, gate bias *V_GS_* = 2.0 V; (**b**) *W_NW_* = 10 nm, *V_GS_* = 2.5 V; (**c**) *W_NW_* = 20 nm, *V_GS_* = 2.5 V; (**d**) *W_NW_* = 25 nm, *V_GS_* = 2.5 V.

To probe the root cause of this undiscovered phenomenon, various curve fittings were performed. We found that the superlinear characteristics can be best plotted with the Poole–Frenkel equation [16,17] (see Figure 3). Under an electric field *E*, the classical Poole–Frenkel emission current density over an insulator is given by

(1) $$J_{PF}=AE\exp\left(\frac{-q(\Phi_{B}-\beta_{PF}\sqrt{E})}{kT}\right)$$

where *A* is a proportionality constant, Φ*_B_* is barrier height, and *kT/q* is the thermal voltage. The PF slope is given by

(2) $$\;\beta_{PF}=\sqrt{\frac{q}{\pi \epsilon_{0}\epsilon_{r}}}=\frac{7.589\times 10^{-4}}{\sqrt{\epsilon_{r}}}\;\{\mathrm{eV}\cdot {\left(\mathrm{cm}/V\right)}^{1/2}\}$$

where ε_0_ is the vacuum permittivity, and *ε_r_* is the dielectric constant of the insulator. That is, the PF slope depends only on the dielectric constant.

Note that the Schottky emission can have a similar field dependency, but the field-dependent slope will be doubled. In addition, the Schottky emission has a stronger temperature dependency. The temperature dependency is an effective way to differentiate the Schottky and PF mechanisms. That is the reason for conducting the temperature-dependent I–V measurements in this work. As will be shown later, the observed characteristics do not follow the temperature dependence of the Schottky emission.

To avoid possible inappropriate estimation of the channel electric field and considering that the local electric field may be deviated from the global fields, in Figure 3, we use the log(*I_DS_/V_DS_*) versus *V_DS_*^1/2^ plot instead of log(*I*_DS_/*E*) versus *E*^1/2^ plot, as defined in (1). With this treatment, the slope of ln(*I_DS_/V_DS_*) − *V_DS_*^1/2^ plot is $\beta ’=\beta_{PF}/\left(kT\sqrt{L_{eff}}\right)$. Namely, the PF slope should be divided by the square root of the effective channel length. As shown in Figure 3a,b, the curves are fairly straight lines, and the gate voltage has almost no effect on the characteristics in the low *V_DS_* region; the linear region extends to a large *V_DS_* region as *V_GS_* increases. The plots also show that the characteristics can be divided into two groups: a smaller PF slope group for temperatures smaller than 350 K, and a larger PF slope group for temperatures above 350 K. This implies that two different sets of PF parameters should be used, or that two different conduction mechanisms may have been involved. For *W_NW_* = 20 nm and 25 nm (Figure 3c,d), the PF plots are much different. First, the linear region in the PF plot is much smaller. Second, it appears that the PF slopes should fall into only one group. Lastly, the PF slope and the Y-intercept value increase almost linearly as the temperature increases.

**Figure 4:**
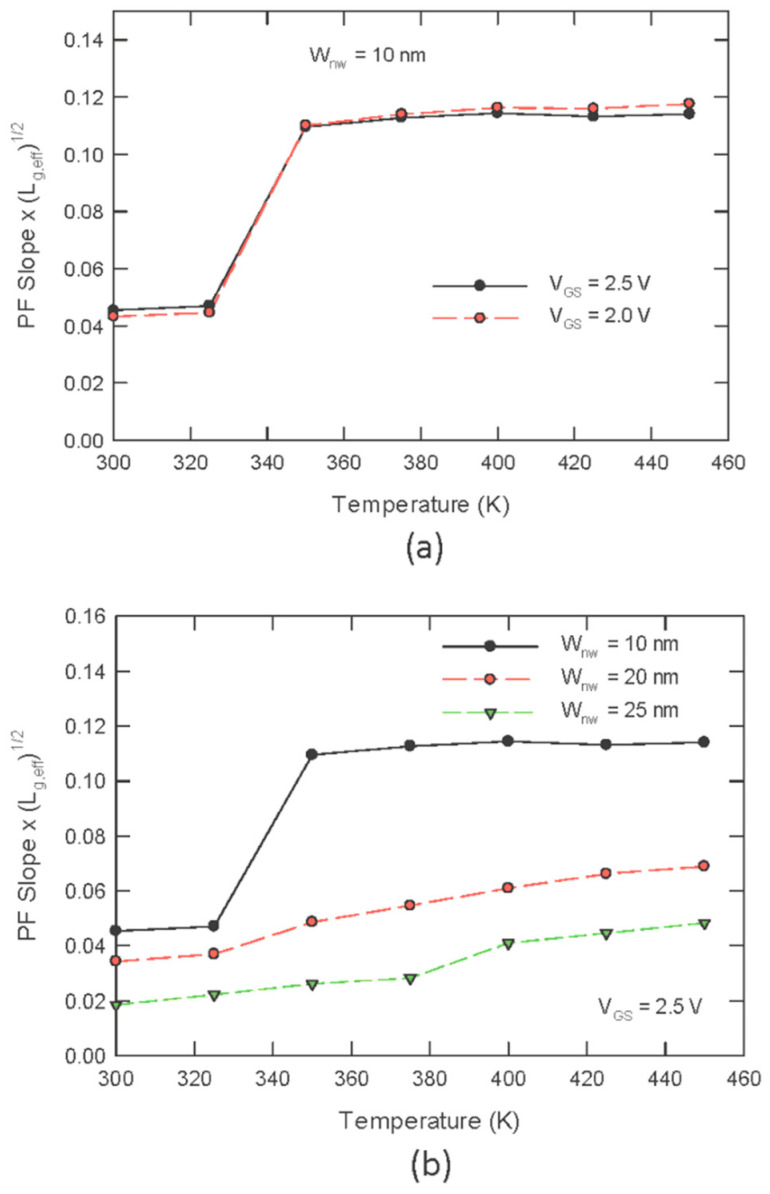
Plot of the PF slope as a function of temperature: (**a**) for narrow nanowires (*W_NW_* = 10 nm), two piecewise regions are observed, and the PF slopes in both regions are fairly temperature independent; (**b**) wider nanowires show an increase in the slope as the temperature increases.

By performing a linear fit on the curves in Figure 4 and multiplying the slopes with $kT\sqrt{L_{eff}}$, the PF slopes can be obtained. The PF slopes only depend on the dielectric constant and are temperature independent, according to (2). For *W_NW_* = 10 nm, we have two piecewise segments of an almost temperature-independent PF slope. Apparently, a different PF slope should result from the different values of the dielectric constants; this is obviously not the case here, however, yet the different slopes should be attributed to the different electric fields generated at different temperature ranges. Although the drain voltages are the same, the local electric field for the case of T < 350 K should be smaller than that for the case of T > 350 K. A higher temperature should result in the completed ionization of the donor atoms and the delocalization of the defect states. Both effects can lead to a higher channel charge density and result in a higher electric field. For *W_NW_* = 20 nm and 25 nm, the values of the PF slopes are much smaller, and they increase almost linearly with the temperature. This indicates that the PF conduction current, in this case, is not the predominant one; the conventional drift current in the inversion channel should contribute a significant portion of the drain current. That is, the PF conduction is more obvious in the 10 nm sample, as the bulk conduction in thin nanowires is much smaller.

Both the PF plots shown in Figure 3 and the temperature independence of the PF slope, as depicted in Figure 4, unambiguously imply that the conduction current in the superlinear region for the *W_NW_* = 10 nm sample should be due to the PF effect. To the best of our knowledge, this is the first report on this phenomenon in the nanowire channel. The obvious PF conduction reported in this work should be a composite effect resulting from (1) the lower substrate doping, (2) higher interface state density, and (3) conducting the characterization at higher temperatures. The physics behind this observation is given in the following.

**Figure 5:**
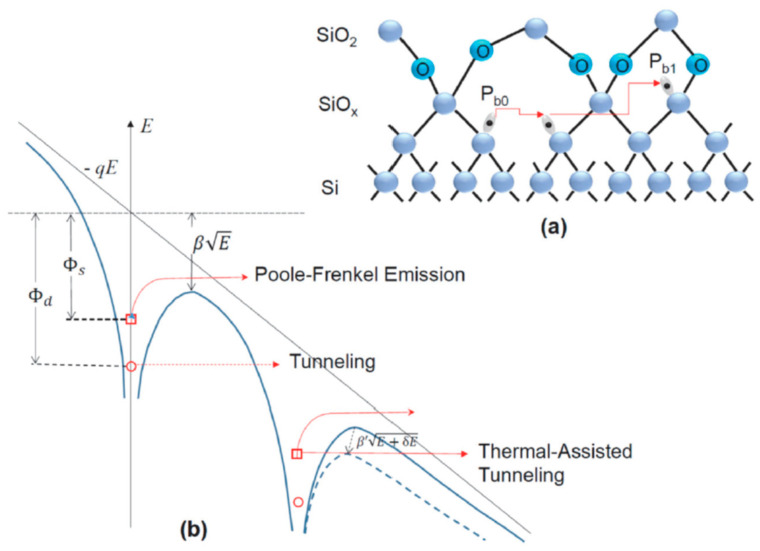
(**a**) Schematic showing the silicon dangling bonds (P_b0_ and P_b1_) at the Si(100) and SiO_2_ interface and the possible charge transport through the neighboring interface states. (**b**) Illustration of the multiple Poole–Frenkel emissions through the interface states along the channel direction. Possible current enhancements due to a high local field, thermal-assisted tunneling, and direct tunneling are also shown.

The observed lateral PF conduction in the nanowire can be attributed to the charge transport between the neighboring interface states. Figure 5a shows the bonding structure at the (100) Si/SiO_2_ interface. At the Si/SiO_2_ interface, there are some threefold-coordinated silicon dangling bonds (Si_3_ ≡ Si), which are called P_b0_ centers. If one of the Si-Si bonds is replaced by Si-O, i.e., O(Si_2_) ≡ Si, it is the P_b1_ center [17,18]. The P_b_ centers contribute the most significant portion of the interface states at the Si/SiO_2_ interface. The density of the surface silicon dangling bonds depends on the crystalline orientation; the silicon (111) surface has the highest amount of P_b0_ centers, and the (100) surface has the lowest. The typical interface state densities at the silicon–silicon thermal oxide interface are in the range of 10^10^ to 10^12^ cm^−2^ eV^−1^ [18]. Other kinds of gate dielectrics, such as silicon oxynitride, silicon nitride, and high-k oxide, have much higher interface state densities than the silicon thermal oxide types. For our nanowire samples, the top surface is (100) and the sidewalls are (110) orientated (see Figure 1). The interface state density in the devices is expected to be on the high side. By measuring the charge pumping current on a gated diode on the same wafer, the interface state density could be calculated by $D_{it}=\frac{1}{qkTWLf}\frac{dI_{cp}}{\mathrm{dln}t_{r}}$, where *I_cp_* is the measured charge pumping current; *f* and *t_r_* are the frequency and rising time of the trapezoidal pulse applied to the gate; and *WL* is the device area. It was found that the interface state density is about 6.5 × 10^11^ eV^−1^cm^−2^. A P_b_ center is an amphoteric defect, and it can capture both electrons and holes. The key effects of the interface states are that they result in a wider transition region between the depletion and accumulation region in the capacitance–voltage (C-V) curves and a threshold voltage shift in the MOS transistors [16]. It has never been reported as a conduction layer along the MOS channel. It was insignificant in the earlier MOS transistors, as both the channel size and the channel inversion charge density were much larger. When all these quantities are reduced to a certain extent, the contribution of the interface state-assisted current conduction can be significant, as revealed in this work.

PF conduction had long been reported as a key conduction mechanism over a dielectric film to the gate (or vertical conduction, in the conventional MOS device perspective [16]). The separations between the well-isolated deep defect states were so large that a large vertical electric field (in several MV/cm) was usually required for PF conduction to occur over the insulator. In this case, the PF conduction was found as the primary or significant component of the drain current in the linear region. For a narrow channel with a low inversion charge density (e.g., <10^12^ cm^2^), the portion of bulk conduction will be rather small. In addition, under a strong gate bias, most of the inversion charges may be localized in the interface states and then involved in the PF conduction or thermally-assisted tunneling along the SiO_2_/Si interface or the interface transition region. The mechanisms for PF-related conduction along the channel direction are illustrated in Figure 5b. PF conduction was not expected to occur at such a low electric field, but the actual electric field involved in the lateral PF conduction along the Si/SiO_2_ interface may be much larger; the interface surface roughness can enhance the local electric field [4]. In addition, we expect that there are more P_b_ centers aligned in the lateral direction, with a smaller separation, which can result in a significant chain PF conduction at lower electric fields.

The P_b0_ center was found to have an energy level of about 0.27 eV under the silicon conduction band [19], and charges trapped on it can be easily delocalized with a certain amount of thermal energy. In addition to the conventional PF emission, the conduction can be enhanced in several ways, as illustrated in Figure 5b. If the separation between the defect states is small enough, direct tunneling is also possible. Meanwhile, if the surface roughness, particularly on the sidewalls, is comparable to the wire size, it will significantly enhance the local electric field, resulting in the barrier lowering [4]. Finally, as the temperature increases, thermally-assisted tunneling is also possible. All the mechanisms combined can lead to the significant current enhancement of the nanowire transistor, especially in the linear region. From the MOS transistor operation point of view, these effects could cause some issues in the degradation of device reliability, excess leakage current, and parameter variability deterioration.

As mentioned previously, the PF conduction is more obvious in the 10 nm sample, as the bulk conduction has been significantly lowered as compared with thicker and wider nanowires. The contribution of PF conduction can be further enhanced by lowering the substrate doping concentration, increasing the interface state density, and increasing the device temperature. If the PF current is comparable to the inversion channel current, the PF enhancement will be more obvious. By increasing the temperature, the effective channel mobility is reduced, making the channel current smaller. Meanwhile, the interface carrier localization probability is reduced, making the interface states active in PF conduction. As the device gate length scales to a few nanometers and the gate dielectric thickness approaches the atomic scale, it is expected that the thickness of the gate dielectric and the surface roughness on the fins in the FinFET structure or on the nanowire in the gate-all-around (GAA) structure will fluctuate. In addition, the gate dielectric–silicon interface and the gate dielectric–metal gate interface could become comparable to the gate dielectric thickness itself. These non-scalable process issues are hard to circumvent and will remain the technological limits for further downsizing of the existing nano CMOS device structures.

## 4. Proposal for Poole–Frenkel Emission MOS Transistor

**Figure 6:**
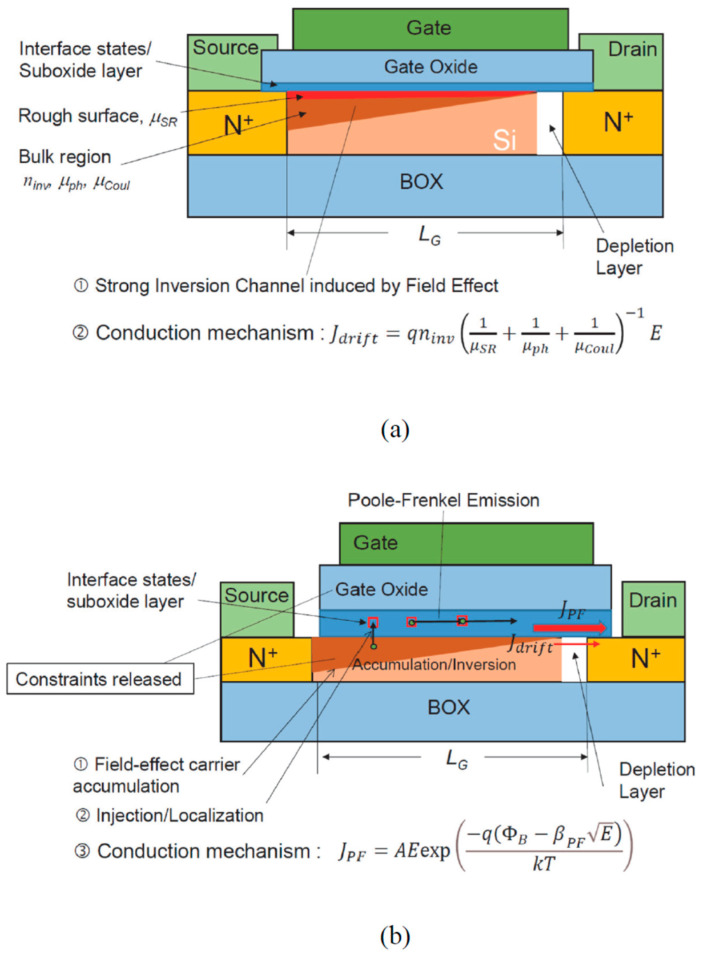
Structural and operation mechanism comparison between the MOSFET (**a**) and the proposed PF-MOS (**b**).

The lateral PF conduction does not necessarily need to be considered as a non-ideal characteristic or as a constraint of the nanowire MOS transistor. Based on the discovery, here we propose a new kind of transistor: the Poole–Frenkel emission MOS transistor, or PF-MOS. The structure, the layout, and thus, its fabrication processes, are essentially the same as those of the current MOS transistors, FinFET, or nanosheet transistors. The major differences in terms of structure and operation principles are illustrated in Figure 6. Figure 6a shows the conventional two-dimensional thin-body MOSFET; the device operation relies on: (1) induction of the channel charge carriers by the field effect (i.e., the vertical field by gate voltage) to maintain channel control; and (2) the drift carrier transport along the inversion channel under the lateral field of the drain bias. With this connection, the gate capacitance should increase accordingly as the device size become smaller. According to the conventional constant field scaling scheme [20], for a 10 nm gate length MOS, the gate oxide EOT should be less than 0.4 nm [21]. However, the actual EOT in the most advanced technology is still in the range of 0.7 to 0.8 nm because of the difficulties in gate dielectric engineering [14]. The drain drift current is governed by channel mobility. When the channel size is large enough, the majority of charge carriers are transported through the near bulk region of the channel, and the channel mobility is mainly governed by the Coulomb and phonon scattering processes, although the surface roughness scattering has never been a negligible factor as an MOS transistor or a surface device. However, as the channel thickness, FinFET, or nanosheet size is reduced to below a deca-nanometer range, the contribution of surface roughness scattering will overwhelm the overall charge transport, resulting in a significant device characteristic degradation [4,12]. Hence, the non-ideal effects resulting from the surface and interface are an unavoidable issue for device downsizing towards the atomic scale. However, the proposal here actually reflects the trend that the ultimate scaling of the device should eventually lead to a truly surface device.

Figure 6b depicts the structure of the ultimate surface device, the lateral Poole–Frenkel emission MOS transistor, or PF-MOS. The structure could be the same as the thin-body fully-depleted MOS, except for the removal of the source/gate oxide overlapping region to avoid the source injection so as to enable a complete turn-off operation of the transistor. For the sake of simplicity, here we illustrate the device in a two-dimensional (2D) view only. PF-MOS favors 3D structures, such as FinFET and nanosheet, because of their large surface area. One of the key advantages of the PF-MOS is that the subnanometer EOT is no longer a must, as it no longer requires a strong inversion channel in the PF-MOS. Instead, an accumulation of a charge density of about 10^12^ to 10^13^ cm^−2^ will be enough for the PF-MOS operation. The PF-MOS involves three different physical mechanisms for operation: (1) to turn on, the gate electric field first induces a certain amount of carriers under the gate oxide; (2) the gate voltage causes in carrier injection, resulting in the charge carriers being localized at the interface states; (3) the lateral field (drain voltage) produces a chain Poole–Frenkel emission along the channel direction. Namely, the conduction channel of PF-MOS is actually the interface layer or suboxide layer between the gate oxide and the silicon substrate. Because the inversion charge under the oxide (channel region of conventional MOS) is low, the drift current via the silicon channel is much smaller than the lateral PF current. As the majority current is governed by PF conduction, the mobility degradation in the ultrathin silicon layer or the small-sized nanowires is no longer an issue. The speed of the device should be much higher than that of the present drift-based current conduction devices. On the contrary, in order to enhance the contribution of PF conduction, the active silicon channel should be reduced as much as possible. That is, in PF-MOS, the constraints of both gate oxide EOT and nanowire size in required by the MOS structure can be relaxed.

Since the PF conduction is via the interface states, we prefer a large surface area, a substrate material with higher surface states such as Si (111) surface, and a higher mismatched, or a larger coordination number difference between Si and the gate dielectric. The characteristics shown in Figure 2a itself, although not yet been optimized, still provide a good demonstration of the potential and characteristics of this kind of transistor. The devices reported here followed the general practice of MOS device fabrication. After the metallization, the device underwent a forming gas (hydrogen-containing inert gas) annealing, which passivated a significant amount of the interface states. Moreover, the gate dielectric used in this work is the thermal oxide, at which the interface state density is relatively low. However, the interface state density should still be higher than the reported nominal Si/SiO_2_ interface values because of the significant contribution of the (110) side wells. To enhance the lateral PF conduction, one can use (111) top and (110) side walls, other types of dielectrics, such as Si_3_N_4_ and high-k metal oxides, and reduce the forming gas annealing.

The PF-MOS has several unique features as compared with the present MOS transistor. First, the drain current of PF-MOS is now governed by field emission instead of drift transport; thus, the power dissipation should be much lower. Second, the drain current is no longer governed by the channel mobility, and the conduction is no longer scaled by the inversion channel; therefore, the technical difficulties of gate oxide EOT scaling and channel mobility degradation can be overcome. Third, the PF conduction can be also enhanced with a shorter channel, and it also favors scaling. At the ultimate scale, further reduction of the nanowire size should have little effect on the PF-MOS. The nanowire size or the channel size can eventually be reduced to a size close to the interface transition layer width in to achieve the best performance of the transistor. Along with the newly proposed PF-MOS, a great deal of investigation can be performed. For a digital application, one would be concerned about the ON and OFF currents. The ON current in the PF-MOS is predominately governed by the PF emission. The PF emission should also affect the OFF current, which is worth a comprehensive investigation.

## 5. Conclusions

By studying the current–voltage characteristics in various nanowire MOS transistors for temperatures ranging from 300 to 450 K, it was found that the I-V characteristics in the conventional ohmic region, i.e., *V_DS_* < *V_GS_* − *V_T_*, are no longer linear; they can be significantly enhanced by the applied drain voltage. The phenomenon is more obvious for narrower nanowires. After careful analysis of the characteristics, we found that the current–voltage relationships strictly follow the Poole–Frenkel emission model. We thus attribute the current conduction to the multiple Poole–Frenkel emission of the localized carriers at the Si/SiO_2_ interface states along the channel direction. This is the first report regarding the lateral Poole–Frenkel current conduction in the nanowire silicon channel. With the new conduction mechanism in place, a new type of MOS device, the Poole–Frenkel emission MOS (PF-MOS) transistor, is proposed. Instead of relying on the inversion layer drift current of the silicon channel, the drain current in the newly proposed PF-MOS is mainly contributed by the lateral Poole–Frenkel emission along the Si/dielectric interface. This structure appears to be promising, as it eliminates the two major technological and physical constraints: the subnanometer gate EOT scaling and channel mobility degradation of MOS device when downsizing towards a small nanometer range.

## Data Availability

No data is available.
